# Treatment and outcome of AIDS-related Kaposi sarcoma in South Africa, Malawi and Zambia: an international comparison

**DOI:** 10.11604/pamj.2017.28.261.11300

**Published:** 2017-11-24

**Authors:** Eliane Rohner, Margaret Kasaro, Susan Citonje Msadabwe-Chikuni, Kathryn Stinson, Zainab Mohamed, Hannock Tweya, Matthias Egger, Julia Bohlius

**Affiliations:** 1 Institute of Social and Preventive Medicine (ISPM), University of Bern, Switzerland; 2 Centre for Infectious Disease Research in Zambia (CIDRZ), Lusaka, Zambia; 3 Cancer Diseases Hospital, Lusaka, Zambia; 4 Khayelitsha ART Program, Médecins Sans Frontières, Cape Town, South Africa; 5 Radiation Oncology, Groote Schuur Hospital, Cape Town, South Africa; 6 Lighthouse Trust at Kamuzu Central Hospital, Lilongwe, Malawi; 7 Centre for Infectious Disease Epidemiology & Research, School of Public Health & Family Medicine, University of Cape Town, Cape Town, South Africa

**Keywords:** Kaposi sarcoma, HIV, Malawi, South Africa, Zambia, treatment

## Abstract

HIV-related Kaposi sarcoma (KS) is common in sub-Saharan Africa, but optimal treatment strategies in resource-limited settings remain unclear. We did a retrospective cohort study of adults diagnosed with KS before initiating antiretroviral therapy (ART) at three ART programs in South Africa, Malawi and Zambia. We extracted data from medical charts at HIV clinics and oncological referral centers and used electronic data from the International epidemiology Databases to Evaluate AIDS Southern Africa. We used descriptive statistics to assess tumor (T) and systemic illness (S) stage and treatment of AIDS-KS patients. Kaplan-Meier analyses were used to assess survival after KS diagnosis. We analyzed data from 57 patients in total (20 from South Africa, 20 from Zambia, 17 from Malawi). Median age at KS diagnosis was 35 years and similar across sites. The percentage of patients with poor risk AIDS-KS (T1S1) was similar in South Africa (25%) and Malawi (24%) and higher in Zambia (45%). All AIDS-KS patients initiated ART at the HIV clinic. For KS care, in South Africa 18 patients (90%) were referred to an oncology department; in Malawi and Zambia most patients were managed by the HIV clinics. In Malawi and South Africa, most AIDS-KS patients received systemic chemotherapy, in Zambia one patient received chemotherapy at the HIV clinic. A year after KS diagnosis, 15 patients (75%) in South Africa, 10 patients (50%) in Zambia, and 8 patients (47%) in Malawi were still alive; another 3 patients (15%) in South Africa, 8 patients (40%) in Zambia and 4 patients (24%) in Malawi were lost to follow-up. Management of AIDS-KS patients varied considerably across sites in Malawi, South Africa and Zambia. We need more reliable survival data for AIDS-KS patients in sub-Saharan Africa before we can assess which treatments and clinical pathways should be adopted in a specific setting.

## Introduction

Kaposi sarcoma (KS) is a common HIV-related malignancy in sub-Saharan Africa (SSA) [1]. While HIV treatment programs have expanded massively over the past decade, resources for oncological care are still very limited in most SSA countries [1]. A recent Cochrane review [2] suggested that, in general, patients with severe HIV-related KS might benefit from a combination of antiretroviral therapy (ART) and chemotherapy; however, optimal management strategies for AIDS-KS patients in resource-limited settings remain unclear. We compared clinical stage, clinical management and outcomes of adult AIDS-KS patients enrolled in ART programs in Malawi, South Africa, and Zambia.

## Methods

In this retrospective cohort study we included adult (aged ≥16 years) AIDS-KS patients diagnosed at three ART programs in South Africa, Malawi and Zambia. Ubuntu Clinic is located in Khayelitsha, a township outside Cape Town. Lighthouse Trust is the largest public ART provider in Malawi, located at the Kamuzu Central Hospital in Lilongwe. The Centre for Infectious Disease Research in Zambia (CIDRZ) is a non-profit organization supporting more than 300 public clinics; we selected three facilities in Lusaka, i.e University Teaching Hospital, Kalinagalinga Clinic, and Kanyama Clinic. All ART programs participate in the International epidemiology Databases to Evaluate AIDS Southern Africa (IeDEA-SA), and have local ethics committees and institutional review board approvals to contribute routine data for IeDEA-SA research projects. Additional approvals were obtained from the Human Research Ethics Committee of the University of Cape Town and the Biomedical Research Ethics Committee of the University of Zambia. Per ART program we aimed to include 20 consecutive patients diagnosed with AIDS-KS before ART initiation from 2011 backwards. We reviewed medical charts at HIV clinics and referral oncology clinics (Groote Schuur Hospital, Cape Town, and Cancer Diseases Hospital, Lusaka) and retrieved data from IeDEA-SA databases. Data was captured electronically using an online tool (REDCap). We assessed tumor (T) and systemic illness (S) stage according to the AIDS Clinical Trials Group (ACTG) system [3]. T and S stages were extracted from charts, if available, and otherwise derived from documented information. T0S0, T0S1 and T1S0 were considered good risk and T1S1 poor risk. CD4 cell count at KS diagnosis was the measurement closest to KS diagnosis (-180/+7 days). Patient and tumor characteristics at baseline and treatments received were analyzed descriptively. Results are presented in percentages, medians and interquartile ranges (IQR). We used Kaplan-Meier analyses to estimate survival after KS diagnosis. Patients were defined as lost to follow-up (LTFU) if there was no contact for ≥6 months prior to the data extraction date. Patients LTFU were censored at last contact date. In additional analyses we combined patients who died with those LTFU. Analyses were done in Stata 13.1 (StataCorp LP).

## Results

**Figure 1:**
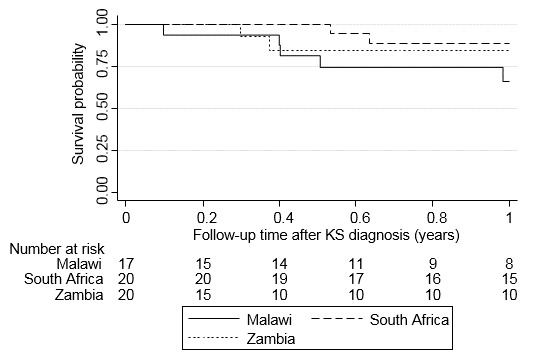
Kaplan-Meier estimates of cumulative survival after KS diagnosis in Malawi, South Africa and Zambia, not including (A) and including (B) loss to follow-up as a failure event

We extracted data for 60 AIDS-KS patients. Three Malawian patients were ineligible; 57 patients were included in the analysis. All patients were diagnosed with AIDS-KS before initiating ART at the HIV clinic. Two KS cases (10%) in South Africa and none in Malawi and Zambia were histologically confirmed. In Malawi and Zambia, most AIDS-KS patients were male; in South Africa, 60% (N = 12) were female (Table 1). Median age at KS diagnosis was 35 years (IQR 24-42 years) and similar across sites. Median CD4 cell count at KS diagnosis was lowest in Zambia (66 cells/µL), followed by Malawi (137 cells/µL) and South Africa (174 cells/µL); the percentage of missing values ranged from 10% to 44% per site. The percentage of patients with poor risk AIDS-KS (T1S1) was higher in Zambia (45%) than in South Africa (25%) and Malawi (24%). Tumors were often located on lower extremities (67%, N = 38) and in the oral cavity (35%, N = 20). Tumor-associated edema was documented in 44% of patients (N = 25). KS lesions on head/neck, trunk, and upper extremities were noted in most South African patients, but rarely in patients from Malawi and Zambia (Table 1). In Zambia, 13 patients (75%) received protease-inhibitors (PI) but none of the first-line ART regimens in South Africa or Malawi included PIs. In South Africa, 18 patients (90%) were referred to an oncology department; in Malawi all treatments were administered at the HIV clinic. In Zambia, only one patient (5%) was referred to the oncology clinic but never appeared there. Most patients in South Africa and Malawi but only one patient in Zambia received chemotherapy (Table 1). In South Africa, all chemotherapy regimens given included ≥2 drugs; most commonly vincristine/bleomycin (60%, N = 12). In Malawi, most patients received vincristine monotherapy (65%, N = 11). Six patients from South Africa (30%), but none from Malawi or Zambia, received radiotherapy. The probability of surviving one year after KS diagnosis was 89% (95% confidence interval (CI) 62%-97%) in South Africa, 84% (50%-96%) in Zambia and 66% (36%-84%) in Malawi (Figure 1). When we included LTFU as a failure event, one-year survival probabilities dropped to 75% (95% CI 50%-89%) in South Africa, 50% (95% CI 27%-69%) in Zambia, and 47% (95% CI 23%-68%) in Malawi.

**Table 1:** Demographics, disease characteristics and treatments of included AIDS-KS patients

	South Africa N (%)	Malawi N (%)	Zambia N (%)
**All patients**	20 (100%)	17 (100%)	20 (100%)
**Gender**			
Female	12 (60%)	4 (24%)	8 (40%)
Male	8 (40%)	13 (76%)	12 (60%)
**Age at KS diagnosis [years]**			
Median (IQR)	34 (29-42)	36 (31-45)	34 (27-39)
**CD4 count at KS diagnosis [cells/µL]**			
Median (IQR)	174 (126-233)	137 (118-334)	66 (23-243)
Missing	2 (10%)	8 (44%)	5 (25%)
**ACTG stage at KS diagnosis**			
T0S0	2 (10%)	1 (6%)	2 (10%)
T0S1	0	0	5 (25%)
T1S0	10 (50%)	11 (65%)	0
T1S1	5 (25%)	4 (24%)	9 (45%)
Missing	3 (15%)	1 (6%)	4 (20%)
**KS location**			
Oral	8 (40%)	4 (24%)	8 (40%)
Head/neck	12 (60%)	0	3 (15%)
Trunk	11 (55%)	1 (6%)	2 (10%)
Upper extremity	10 (50%)	2 (12%)	1 (5%)
Lower extremity	15 (75%)	15 (88%)	8 (40%)
Tumor-associated edema	12 (60%)	8 (47%)	5 (25%)
Visceral (suspected)	2 (10%)	2 (11%)	1 (5%)
Unclear/unknown	2 (10%)	1 (6%)	4 (20%)
**Systemic chemotherapy**			
Yes	18 (90%)	17 (100%)	1 (5%)
No	0	0	18 (90%)
Unclear/unknown	2 (10%)	0	1 (5%)
**Chemotherapy regimen**			
Vincristine	0	11 (65%)	0
Vincristine/Bleomycin	12 (60%)	5 (29%)	0
Other two-drug combination chemotherapy	2 (10%)	0	1 (5%)
Doxorubicin/Bleomycin/Vincristine	4 (20%)	1 (6%)	0
None	0	0	18 (90%)
Unclear/unknown	2 (10%)	0	1 (5%)
**Radiotherapy**			
Yes	6 (30%)	0	0
No	12 (60%)	17 (100%)	20 (100%)
Unclear/unknown	2 (10%)	0	0

ACTG, AIDS Clinical Trials Group; ART, antiretroviral therapy; IQR, interquartile range; KS, Kaposi sarcoma; N, number

## Discussion

For this international comparison, we deliberately identified patients at HIV clinics and followed their path through the health care system. We extracted data from charts of HIV clinics and oncology departments. The retrospective design ensured that we captured routine conditions, and that our study had no effect on clinical management. Clinical management of AIDS-KS patients varied across sites and countries in SSA. While South Africa offered combination chemotherapy at specialized oncology departments, Malawi and Zambia usually managed AIDS-KS patients at the ART programs. One year after KS diagnosis 75% of patients in South Africa and less than 50% of patients in Malawi and Zambia were alive and in care. Some limitations need to be addressed. Several patients were LTFU early after KS diagnosis, and generally, patients LTFU are more likely to have died than patients who remained in care [4]. To assess the impact of differential LTFU on the survival probabilities we performed sensitivity analyses including LTFU as a failure event. The extent of data recorded in medical charts varied between sites and relevant study data was often not available. For example, we could not assess KS response to treatment because location, number and extent of KS lesions were not documented in detail. In general, KS-specific documentation was more comprehensive in oncology departments than at HIV clinics, which might have caused us to underestimate clinical KS stage in patients not referred to oncology departments. The AIDS-KS patient load and calendar periods covered varied between sites, and this reduced comparability across sites. As patient populations and treatment approaches differ between ART programs, our findings are unlikely to be representative of all ART programs in Malawi, South Africa, and Zambia. Many patients were diagnosed in advanced AIDS-KS stage; in line with results from other studies in the region [5, 6]. In Malawi, all patients received chemotherapy, as described in a previous Lighthouse Trust study [7].

We found that 90% of patients from Khayelitsha (Cape Town) received chemotherapy, while an earlier study reported that only 29% received chemotherapy [5]. This difference is likely due to improved service delivery over time. To our knowledge, published data on routine KS treatment at ART programs in Zambia are not available. Our one-year survival estimate from Lighthouse Trust was lower than a recent estimate from a specialized KS clinic in Malawi [6], perhaps because different chemotherapy regimens were used, and perhaps because more specialized oncological care was available at the KS clinic. Our survival estimate for South Africa was in line with a previous study [8]. Another study from Khayelitsha [5], which recruited patients earlier than we did (2001-2007 vs. 2005-2011), had shown a lower survival estimate at one year (60%). This might be explained by the higher proportion of patients in our study who received ART and chemotherapy, but also by temporal survival improvements in AIDS-KS patients. When ART coverage was low in SSA, most AIDS-KS patients were diagnosed in advanced KS stage and died a few months later. Since 2004, ART availability has increased substantially in Southern Africa, and efforts have been made to improve HIV testing and linkage to care. AIDS-KS patients should now be diagnosed in earlier KS stage. However, our and other studies have shown that in SAA, patients are still diagnosed in advanced KS stages [5,6]. In addition to HIV treatment, KS patients are likely to benefit from specialized oncology care. However, data from SSA are scarce and findings from high-income countries cannot be extrapolated to African settings. Survival after KS diagnosis is an essential measure to assess and compare treatment outcomes in AIDS-KS patients. However, survival data are prone to bias if LTFU is substantial [9]. AIDS-KS patients are more likely to be LTFU than HIV-positive patients without KS [10], so these patients might require dedicated tracking efforts to obtain reliable survival estimates.

## Conclusion

Despite increasing ART availability, many patients are still diagnosed in advanced AIDS-KS stage in SSA. Management of AIDS-KS patients varied considerably across sites in Malawi, South Africa, and Zambia, and it is unclear which treatment procedures and clinical paths should be adopted in which setting. Better survival data is needed to reliably assess outcomes across different standards of care.

### What is known about this topic

- Kaposi sarcoma is a common HIV-related malignancy in sub-Saharan Africa;
- Many patients in sub-Saharan Africa are diagnosed in advanced KS stages;
- Optimal management strategies for AIDS-KS patients in resource-limited settings remain unclear.

### What this study adds

- Management of AIDS-KS patients varied considerably across sites in Malawi, South Africa, and Zambia;
- The majority of patients in South Africa were referred to oncology departments for KS care, whereas in Malawi and Zambia AIDS-KS patients were usually managed by the ART programs;
- Most patients in Malawi and South Africa, but only one patient in Zambia received chemotherapy.

## Competing interests

The authors declare no competing interests.

## References

[1] Kingham TP, Alatise OI, Vanderpuye V, Casper C, Abantanga FA, Kamara TB, Olopade OI, Habeebu M, Abdulkareem FB, Denny L (2013). Treatment of cancer in sub-Saharan Africa. Lancet Oncol..

[2] Gbabe OF, Okwundu CI, Dedicoat M, Freeman EE (2014). Treatment of severe or progressive Kaposi's sarcoma in HIV-infected adults. Cochrane Database Syst Rev..

[3] Krown SE, Metroka C, Wernz JC (1989). Kaposi's sarcoma in the acquired immune deficiency syndrome: a proposal for uniform evaluation, response and staging criteria: AIDS Clinical Trials Group Oncology Committee. J Clin Oncol..

[4] Brinkhof MWG, Pujades-Rodriguez M, Egger M (2009). Mortality of patients lost to follow-up in antiretroviral treatment programmes in resource-limited settings: systematic review and meta-analysis. PLoS One..

[5] Chu KM, Mahlangeni G, Swannet S, Ford NP, Boulle A, Van Cutsem G (2010). AIDS-associated Kaposi's sarcoma is linked to advanced disease and high mortality in a primary care HIV programme in South Africa. J Int AIDS Soc..

[6] Herce ME, Kalanga N, Wroe EB, Keck JW, Chingoli F, Tengatenga L, Gopal S, Phiri A, Mailosi B, Bazile J, Beste JA, Elmore SN, Crocker JT, Rigodon J (2015). Excellent clinical outcomes and retention in care for adults with HIV-associated Kaposi sarcoma treated with systemic chemotherapy and integrated antiretroviral therapy in rural Malawi. J Int AIDS Soc..

[7] Mwafongo Aa, Rosenberg NE, Ng'ambi W, Werner AB, Garneau WM, Gumulira J, Phiri S, Hosseinipour MC (2014). Treatment outcomes of AIDS-associated Kaposi's sarcoma under a routine antiretroviral therapy program in Lilongwe, Malawi: Bleomycin/vincristine compared to vincristine monotherapy. PLoS One..

[8] Mosam A, Shaik F, Uldrick TS, Esterhuizen T, Friedland GH, Scadden DT, Aboobaker J, Coovadia HM (2012). A randomized controlled trial of highly active antiretroviral therapy versus highly active antiretroviral therapy and chemotherapy in therapy-naive patients with HIV-associated Kaposi sarcoma in South Africa. J Acquir Immune Defic Syndr..

[9] Freeman E, Semeere A, Wenger M, Bwana M, Asirwa FC, Busakhala N, Oga E, Jedy-Agba E, Kwaghe V, Iregbu K, Jaquet A, Dabis F, Yumo HA, Dusingize JC, Bangsberg D, Anastos K, Phiri S, Bohlius J, Egger M, Yiannoutsos C, Wools-Kaloustian K, Martin J (2016). Pitfalls of practicing cancer epidemiology in resource-limited settings: the case of survival and loss to follow-up after a diagnosis of Kaposi's sarcoma in five countries across sub-Saharan Africa. BMC Cancer..

[10] Maskew M, Fox MP, van Cutsem G, Chu K, Macphail P, Boulle A, Egger M (2013). Treatment response and mortality among patients starting antiretroviral therapy with and without Kaposi sarcoma: a cohort study. PLoS One..

